# Supplementary material for: A UMLS-based spell checker for natural language processing in vaccine safety
Source: BMC Med Inform Decis Mak. 2007 Feb 12;7:3. doi: 10.1186/1472-6947-7-3 (PMC1805499; doi:10.1186/1472-6947-7-3)
Supplement: Additional file 13 — RAP application directory. Sets up directory for RAP (RDF application for PHP) source code [file 1472-6947-7-3-S13.gz › rap/doc/phpdoc/syntax/RdfSerializer.html]

xml version="1.0" encoding="iso-8859-1"?


Docs For Class RdfSerializer


## Class RdfSerializer

Description

Description |
Vars (details)
| Methods (details)

An RDF seralizer.

Seralizes models to RDF syntax. It supports the xml:base, xml:lang, rdf:datatype and rdf:nodeID directive. You can choose between different output syntaxes by using the configuration methods or changing the configuration default values in constants.php. This class is based on the java class edu.unika.aifb.rdf.api.syntax.RDFSerializer by Boris Motik.

History:

- 12-14-2004 : bug in function serialize() fixed by S�ren Auer(auer@informatik.uni-leipzig.de)
- 12-06-2004 : improved namespace handling added (tobias.gauss@web.de)
- 01-06-2004 : Empty model bug fixed.
- 03-25-2004 : Bug in saveAs() fixed by inkel(inkel-php@f14web.com.ar)
- 03-21-2004 : Support for xml default namespace added
  Methods changed: serialize(), writeNamespaceDeclarations(), getElementText()
- 11-17-2003 : Support for XMLLiterals added.
- 07-27-2003 : Functions addapted to the new class tree (MemModel extends Model)
- 07-24-2003 : Bug in writeAbsoluteResourceReference() fixed by Paul Cowles(paul@semaview.com)
- 02-21-2003 : saveAs method added.
- 02-12-2003 : rdf:type and bNode reference bug fixed.
- 01-15-2003 : pass-by-reference bug fixed.
- 01-10-2003 : rdf:datatype and $useAttributes bug fixed.
- 12-04-2002 : Added support for rdf:datatype (writeContentStatements())
- 12-04-2002 : Added rdf:nodeID attribute for blank nodes (writeSubjectURI, writeResourceReference)
- 10-03-2002 : Bug in collectNamespaces() fixed.
- 09-15-2002 : First version of this class.

- access: public
- author: Chris Bizer <chris@bizer.de>, Boris Motik <motik@fzi.de>, Daniel Westphal <dawe@gmx.de>, Leandro Mariano Lopez <llopez@xinergiaargentina.com>
- version: V0.9.1

Located in /syntax/RdfSerializer.php (line 43)

```
Object
   |
   --RdfSerializer
```

Variable Summary

Description |
Vars (details)
|
Methods (details)

mixed
$m\_attributeStatements

mixed
$m\_baseURI

mixed
$m\_contentStatements

mixed
$m\_currentSubject

mixed
$m\_defaultNamespaces

mixed
$m\_groupTypeStatement

mixed
$m\_namespaces

mixed
$m\_nextAutomaticPrefixIndex

mixed
$m\_out

mixed
$m\_rdfAboutElementText

mixed
$m\_rdfIDElementText

mixed
$m\_rdfResourceElementText

mixed
$m\_statements

mixed
$rdf\_qnames

mixed
$rdf\_qname\_prefix

mixed
$sort\_model

mixed
$use\_attributes

mixed
$use\_entities

mixed
$use\_xml\_declaration

Method Summary

Description |
Vars (details)
|
Methods (details)

RdfSerializer
RdfSerializer
()

void
addNamespacePrefix
(String $prefix, String $namespace)

void
configSortModel
(boolean $bool)

void
configUseAttributes
(boolean $bool)

void
configUseEntities
(boolean $bool)

void
configUseQnames
(boolean $bool)

void
configUseXmlDeclaration
(boolean $bool)

boolean
saveAs
(object MemModel &$model, mixed $filename, [String $encoding = DEFAULT\_ENCODING])

string
&serialize
(object MemModel &$model, [mixed $xml\_default\_namespace = NULL], [String $encoding = DEFAULT\_ENCODING])

Variables

Description |
Vars (details)
|
Methods (details)

mixed
$m\_attributeStatements
= array() (line 64)

mixed
$m\_baseURI
(line 57)

mixed
$m\_contentStatements
= array() (line 65)

mixed
$m\_currentSubject
(line 59)

mixed
$m\_defaultNamespaces
= array() (line 53)

mixed
$m\_groupTypeStatement
(line 63)

mixed
$m\_namespaces
= array() (line 54)

mixed
$m\_nextAutomaticPrefixIndex
(line 55)

mixed
$m\_out
(line 56)

mixed
$m\_rdfAboutElementText
(line 61)

mixed
$m\_rdfIDElementText
(line 60)

mixed
$m\_rdfResourceElementText
(line 62)

mixed
$m\_statements
= array() (line 58)

mixed
$rdf\_qnames
(line 49)

mixed
$rdf\_qname\_prefix
(line 66)

mixed
$sort\_model
(line 48)

mixed
$use\_attributes
(line 47)

mixed
$use\_entities
(line 46)

mixed
$use\_xml\_declaration
(line 50)

Methods

Description |
Vars (details)
Methods (details)

Constructor RdfSerializer (line 73)

Constructor

- access: public

RdfSerializer

RdfSerializer
()

addNamespacePrefix (line 163)

Adds a new prefix/namespace combination.

- access: public

void

addNamespacePrefix
(String $prefix, String $namespace)

- String
  $prefix
- String
  $namespace

configSortModel (line 99)

Serializer congiguration: Sort Model Flag if the serializer should sort the model by subject before serializing.

TRUE makes the RDF code more compact. TRUE is default. Default can be changed in constants.php.

- access: public

void

configSortModel
(boolean $bool)

- boolean
  $bool

configUseAttributes (line 125)

Serializer congiguration: Use Attributes Flag if the serializer should serialize triples as XML attributes where possible.

TRUE makes the RDF code more compact. FALSE is default. Default can be changed in constants.php.

- access: public

void

configUseAttributes
(boolean $bool)

- boolean
  $bool

configUseEntities (line 112)

Serializer congiguration: Use Entities Flag if the serializer should use entities for URIs.

TRUE makes the RDF code more compact. FALSE is default. Default can be changed in constants.php.

- access: public

void

configUseEntities
(boolean $bool)

- boolean
  $bool

configUseQnames (line 138)

Serializer congiguration: Use Qnames Flag if the serializer should use qualified names for RDF reserved words.

TRUE makes the RDF code more compact. TRUE is default. Default can be changed in constants.php.

- access: public

void

configUseQnames
(boolean $bool)

- boolean
  $bool

configUseXmlDeclaration (line 151)

Serializer congiguration: Use XML Declaration Flag if the serializer should start documents with the xml declaration <?xml version="1.0" encoding="UTF-8" ?>.

TRUE is default. Default can be changed in constants.php.

- access: public

void

configUseXmlDeclaration
(boolean $bool)

- boolean
  $bool

saveAs (line 307)

Serializes a model and saves it into a file.

Returns FALSE if the model couldn't be saved to the file.

- access: public

boolean

saveAs
(object MemModel &$model, mixed $filename, [String $encoding = DEFAULT\_ENCODING])

- object MemModel
  $model
- String
  $encoding

serialize (line 181)

Serializes a model to RDF syntax.

RDF syntax can be changed by config\_use\_attributes($boolean), config\_use\_entities($boolean), config\_sort\_model($boolean). NOTE: There is only one default namespace allowed within an XML document. Therefore if SER\_RDF\_QNAMES in constants.php is set to FALSE and you pass another $xml\_default\_namespace as parameter, the model will be serialized as if SER\_RDF\_QNAMES were set to TRUE.

- access: public

string

&serialize
(object MemModel &$model, [mixed $xml\_default\_namespace = NULL], [String $encoding = DEFAULT\_ENCODING])

- object MemModel
  $model
- String
  $encoding

#### Inherited Methods


Inherited From Object

> Object::toString()

Documentation generated on Thu, 7 Jul 2005 13:43:03 +0200 by phpDocumentor 1.3.0RC3
